# Analgesic effects of sufentanil in combination with flurbiprofen axetil and dexmedetomidine after open gastrointestinal tumor surgery: a retrospective study

**DOI:** 10.1186/s12871-022-01670-0

**Published:** 2022-04-29

**Authors:** Fei Liu, Ting-Ting Li, Lu Yin, Jin Huang, Yan-Jun Chen, Liu-Lin Xiong, Ting-Hua Wang

**Affiliations:** 1 Department of Anesthesiology, Institute of Neurological Disease, West China Hospital, Sichuan University, No. 37 Guoxue lane, Chengdu, 610041 Sichuan China; 2 Department of Neurosurgery, First Affiliated Hospital of Kunming Medical University, Kunming, 650000 Yunnan China; 3 Department of Anesthesiology, Affiliated Hospital of Zunyi Medical University, Zunyi, 563000 Guizhou China

**Keywords:** Sufentanil, Flurbiprofen axetil, Dexmedetomidine, Multimodal analgesia, Patient-controlled analgesia, Open gastrointestinal tumor surgery

## Abstract

**Background:**

To investigated the effects of sufentanil in combination with flurbiprofen axetil and dexmedetomidine for patient-controlled intravenous analgesia (PCIA) on patients after open gastrointestinal tumor surgery, and compared this combination with traditional PCIA with pure opioids or epidural analgesia (PCEA).

**Methods:**

Patients (*n* = 640) who underwent open gastrointestinal tumor surgery and received patient-controlled analgesia (PCA) were included. According to the type of PCA, patients were assigned to three groups: MPCIA (PCIA with sufentanil, flurbiprofen axetil, dexmedetomidine and metoclopramide), OPCIA (PCIA with sufentanil, tramadol and metoclopramide) and PCEA group (PCEA with sufentanil and ropivacaine). The characteristics of patients, intraoperative use of analgesics, postoperative visual analogue scale (VAS), postoperative adverse reactions and postoperative recovery were collected. The primary outcome was postoperative VAS score. One-way ANOVA, Kruskal-Wallis H test, Fisher exact probability method, and binary logistic regression analysis were used for analysis.

**Results:**

There were no significant differences in the characteristics of patients, operation time, tumor site and the use of postoperative rescue analgesics among the groups. In the first two days after open gastrointestinal tumor surgery, the VAS (expressed by median and interquartile range) of MPCIA (24^th^ h, resting: 1,1; movement: 3,2. 48^th^ h, resting: 0,1; movement: 2,1.) and PCEA (24^th^ h, resting: 0,1; movement: 2,1. 48^th^ h, resting: 0,1; movement: 2,2.) groups were significantly lower than those of OPCIA group (24^th^ h, resting: 2.5,2; movement: 4,2. 48^th^ h, resting: 1.5,1.75; movement: 3,1.) (all *p* <  0.01). The incidence of postoperative nausea and vomiting in MPCIA group was 13.6% on the first day after surgery, which was significantly higher than that in PCEA group. There was no significant difference in the incidence of other postoperative adverse events. Higher intraoperative sufentanil dosage (OR (95%CI) = 1.017 (1.002–1.031), *p* = 0.021), lower body mass index (OR (95%CI) = 2.081 (1.059–4.089), *p* = 0.033), and tumor location above duodenum (OR (95%CI) = 2.280 (1.445–3.596), *p* <  0.001) were associated with poor postoperative analgesia.

**Conclusions:**

The analgesic effects of PCIA with sufentanil in combination with flurbiprofen axetil and dexmedetomidine on postoperative analgesia was better than that of traditional pure opioids PCIA, and similar with that of PCEA.

**Supplementary Information:**

The online version contains supplementary material available at 10.1186/s12871-022-01670-0.

## Background

The patients underwent open gastrointestinal tumor surgery often experienced intensity pain after surgery. Poor postoperative pain control can lead to multi-system disorders, affecting patients’ activities and recovery of physiological functions, reducing postoperative satisfaction and quality of life [1]. Patient-controlled analgesia (PCA), was a common method of postoperative analgesia. In the wake of serious and persistent concern on the opioid epidemic in the USA, there has been a recent renewal of interest in non-opioid alternatives or adjuncts in controlling postoperative pain, often in the context of multimodal analgesia [2].

Sufentanil, a potent α-1 agonistic opioid, was widely used in clinical practice because of its powerful analgesic action [3]. Dexmedetomidine, a highly selective agonist of α2-adrenergic receptor, had the effects of analgesia, sedation and opioids-like protection [4]. Some study had proved dexmedetomidine combined with sufentanil in the patient-controlled intravenous analgesia (PCIA) provided better analgesic effects, and improved the early postoperative cognitive function [5–7]. Flurbiprofen axetil was a commonly prescribed agent to relieve the pain [8, 9]. The combination of dexmedetomidine and flurbiprofen axetil could reduce the pain intensity, restlessness and cognitive dysfunction [10]. And the combination of flurbiprofen axetil and sufentanil in the postoperative PCIA could reduce postoperative VAS scores of patients with colorectal cancer surgery [11, 12]. Sufentanil combined with flurbiprofen axetil or dexmedetomidine has been widely studied in postoperative analgesia, but the comparison of analgesia effect of the combination of these three in postoperative PCIA was rarely reported. Moreover, it has not been cleared that the comparison analgesic effect and related adverse reactions among the multi analgesics PCIA, the traditional opioid-PCIA and patient-controlled epidural analgesia (PCEA). Since 2017, some patients in West China Hospital have started to use the multimodal analgesia plan of sufentanil in combination with dexmedetomidine and flurbiprofen axetil for PCIA. So, in this study reviewed the postoperative pain scores and recovery of patients who received different analgesia methods, to serve as a reference for the selection of clinical analgesia methods in the future.

## Methods

### Patients and group

Patients who received patient-controlled analgesia (PCA) for postoperative pain management between October 2017 and July 2018 were included in this study. Then, patients were divided into three groups according to the type of PCA they received: multimodal PCIA (MPCIA) group, traditional pure opioids PCIA (OPCIA) group and epidural analgesia (PCEA) group. All patients underwent open gastrointestinal tumor surgery under endotracheal intubation and general anesthesia. PCA was used for postoperative analgesia at the end of surgery.

The formulation of PCA used in MPCIA group was sufentanil (2 μg/kg) + flurbiprofen axetil (400 mg) + dexmedetomidine (200 μg) + metoclopramide (60 mg) + appropriate normal saline, a total of 200 ml analgesic solution. The formulation of PCA used in OPCIA group was sufentanil (200 μg) + tramadol (1000 mg) + metoclopramide (60 mg) + appropriate normal saline, a total of 200 ml analgesic solution. MPCIA group and OPCIA group were given analgesic solution by intravenous infusion at the end of operation. The background dose was 2 ml/h, and the additional dose of automatic analgesia was 0.5 ml/15 min. The formulation of PCA in PCEA group was sufentanil (100 μg) + ropivacaine (300 mg) + appropriate normal saline, a total of 200 ml analgesic solution. Patients in the PCEA group received epidural catheterization through the thoracolumbar space (Gastric surgery: T9–10, ascending/descending/transverse colon surgery: T10–11, sigmoid/rectum surgery: T12-L1) before surgery under local anesthesia. After the operation, analgesic fluid was administered through the epidural space. The background dose was 4–8 ml/h, and the additional dose of automatic analgesia was 4 ml/20 min.

Inclusion criteria: 1) Patients underwent open gastrointestinal tumor surgery; 2) Surgery were performed under general anesthesia with endotracheal intubation; 3) PCA was used for postoperative analgesia; 4) Age 18 years and above; 5) American Society of Anesthesiologists Classification: I-III level.

Exclusion criteria: 1) Patients with severe drug allergy; 2) Patients with coronary heart disease or coronary stents; 3) Patients with severe heart failure; 4) Patients with severe liver, kidney or blood system disorders; 5) Patients with prior history of severe hemorrhagic gastrointestinal tract disease, such as ulcerative hydroenteritis, Crohn’s disease; 6) Patients with incomplete postoperative analgesia information; 7) Only enterostomy or combined with multiple site surgery.

### Analgesia strategies

Analgesia plan was a choice made by anesthesiologist according to their own experience and patients’ wishes before the operation. Due to the introduction of the concept of acute pain service team (APS) [13], PCA used by all patients in the medical unit was provided by the same team.

All cases were treated with sufentanil during the operation for analgesia. About half an hour before the end of surgery, tramadol 100 mg was administered intravenously for preemptive postoperative analgesia. Immediately after the operation, PCA was administered via vein (MPCIA group and OPCIA group) or epidural space (PCEA group) for postoperative analgesia. When the patient’s pain could not be alleviated (visual analogue scale (VAS) score > 3) after two consecutive given additional dose by PCA, rescue analgesics were used for analgesia.

### Sources of information

Data were collected from patients’ medical records. The characteristics of patient (gender, age, height, weight and body mass index (BMI)), tumor location and time of operation were included to analyze the comparability between groups.

The primary outcomes: The VAS (A range of 0 to 10. A score of 0 means no pain and a score of 10 means unbearable pain) when patients at rest and movement state at 24 hour (h) and 48 h after surgery;

The secondary outcomes: 1) Intraoperative dose of sufentanil (ug); 2) Use of rescue analgesics at 24 h after surgery; 3) Incidence of postoperative nausea and vomiting (PONV) at 24 h and 48 h after surgery; 4) Time from the end of surgery to the resumption of activity (h), including first anal exhaust, first drinking water, first off-bed activity, remove urinary catheter, abdominal drainage tube and gastric tube; 5) Length of postoperative hospital stay (days, d); 6) Incidence of adverse events, including but not limited to reoperation, anastomotic fistula, peptic ulcer, gastrointestinal bleeding, and cardiovascular disease.

### Statistical analysis

According to the primary outcome of the study, since the pain difference of patients in resting state was smaller than that in movement state, this study included the VAS score of resting state at 24 h after surgery for sample size estimation. At 24 h after surgery, in the patient’s resting state, the postoperative VAS of the previous OPCIA group and MPCIA group were 1.3 ± 1.1 and 2.4 ± 1.3 respectively. So, the minimum sample size required for each group in this study is about 21 cases (α = 0.05, 1 - β = 80%) [14]. Considering that this study was a retrospective analysis, we collected as many cases as possible, and each group of cases met the requirements for sample size.

All data was entered into SPSS version 25.0 for statistical analysis. If the quantitative data were normally distributed, one-way ANOVA was used for analysis and expressed by mean ± standard deviation. For those with positive results on one-way ANOVA, multiple comparisons were performed by Bonferroni. If the quantitative data were not normally distributed, Kruskal-Wallis H test was used and expressed by median (Md) and interquartile range (IQR). The categorical variables were performed using Fisher exact probability method and expressed as number with percentage. The correlation (expressed by correlation coefficient, *r*) between the postoperative VAS scores at 24 h and intraoperative dose of sufentanil was performed by using the Spearman. According to whether the VAS exceeds 3 scores, the VAS in the movement state at 24 h after surgery were divided into two groups. The factors leading to poor postoperative analgesia were analyzed by binary logistic regression analysis, and the results were expressed by odds ratio (OR) value and 95% confidence interval (95% CI). *p* < 0.05 was considered as statistically significant.

Furthermore, propensity score matching (PSM) function of SPSS 25.0 was used to carry out 1:1 propensity score matching for gender, age, height, weight, BMI, tumor location, time of surgery and intraoperative sufentanil dosage in the groups (MPCIA group and OPCIA group, MPCIA group and PCEA group). The caliper value was set as 0.02 to obtain covariate balanced samples between groups. Sensitivity analysis was then performed for the groups.

## Results

**Fig. 1:**
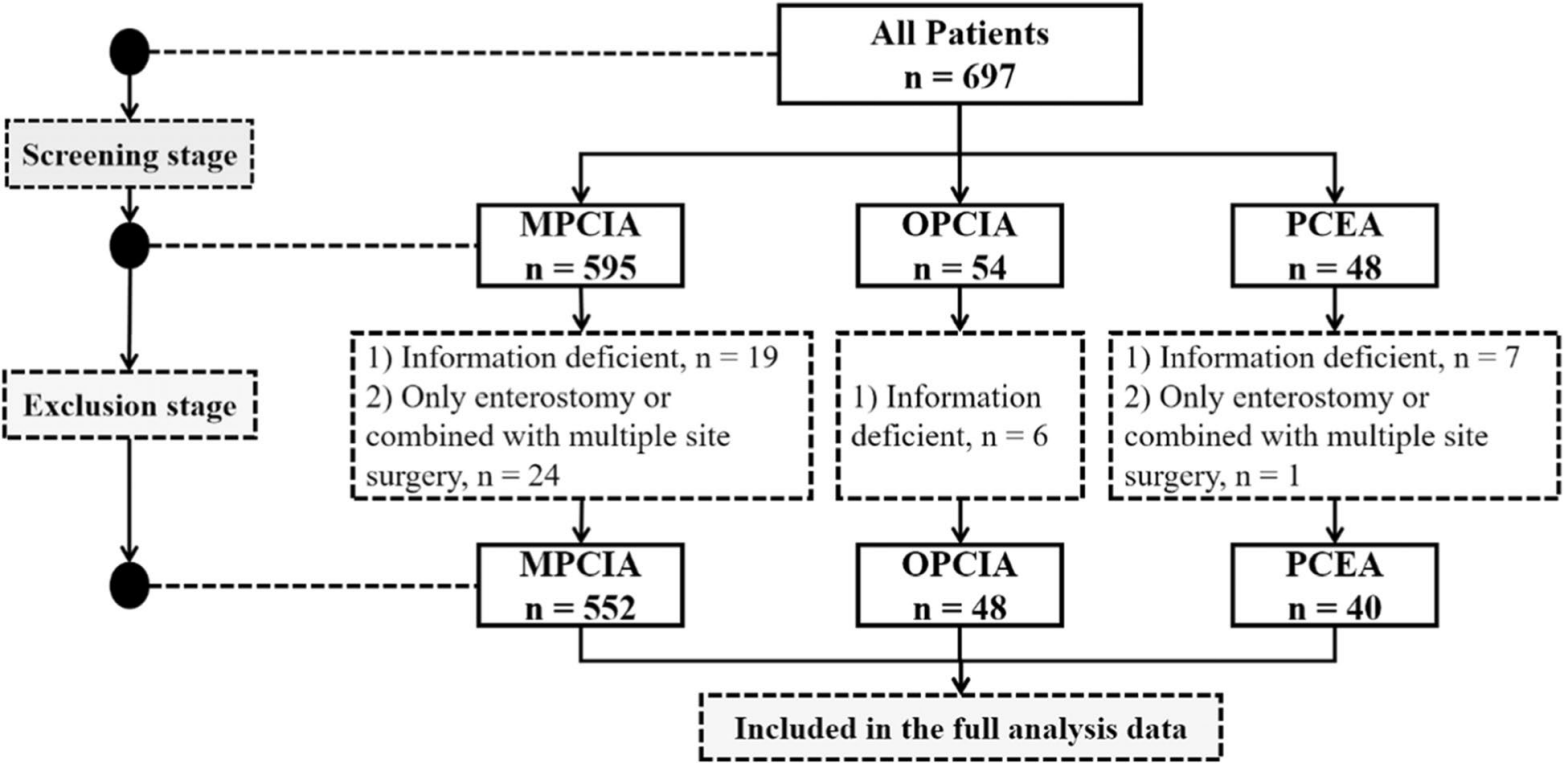
Flowchart of included patients

According to inclusion and exclusion criteria, a total of 640 patients with gastrointestinal tumor were finally included for analysis (Fig. 1). Of these, 387 cases were male and 253 cases were female. The mean age of the overall patients was 58.2 ± 12.1 years (in the range of 19 to 84 years), and 36.1% were older than 65 years. The mean of BMI in the all patients was 22.9 ± 3.1 kg/m^2^ (in the range of 15.6 and 39 kg/m^2^). There were 30.0% overweight (a BMI of 24.0–27.99 kg/m^2^) patients and 5.1% obese (BMI greater than 28.0 kg/m^2^) patients. All patients underwent open gastrointestinal tumor surgery under general anesthesia. Among them, 41.7% of tumors were located in duodenum and above (such as gastric cancer), 41.3% in sigmoid colon and below (such as rectal cancer), and 17.0% in between the duodenum and sigmoid colon. After surgery, MPCIA was used in 552 patients, OPCIA in 48 patients, and PCEA in 40 patients for postoperative analgesia.

### Baseline comparison

There were no statistically significant differences in gender (*p* = 0.953), age (*p* = 0.784), age cohorts (*p* > 0.05), height (*p* = 0.805), weight (*p* = 0.227), BMI (*p* = 0.077), BMI cohorts (*p* = 0.061), tumor location (*p* = 0.533) and operation time (*p* = 0.316) among the three groups (Table 1).

**Table 1:** Baseline comparison between the three groups

		ALL *n = 640*	MPCIA *n = 552*	OPCIA *n = 48*	PCEA *n = 40*	Difference [95% CI]			Statistical value	*p* value
						MPCIA vs OPCIA	MPCIA vs PCEA	OPCIA vs PCEA		
Gender, *n(%)*	Male	387 (60.5%)	335 (60.7%)	28 (58.3%)	24 (60.0%)				0.149	0.953
	Female	253 (39.5%)	217 (39.3%)	20 (41.7%)	16 (40.0%)					
Age, *year-old, M ± Std (Min-Max)*		58.2 ± 12.1 (19.0–84.0)	58.3 ± 12.3 (19.0–84.0)	58.7 ± 10.4 (28.0–79.0)	57.0 ± 11.2 (34.0–78.0)	− 0.44 [−4.82,3.94]	1.26 [−3.51,6.03)	1.70 [−4.53,7.94)	0.244	0.784
Age cohorts, *n(%)*	≧ 60 years	318 (49.7%)	276 (50.0%)	23 (47.9%)	19 (47.5%)				0.177	0.926
	≧ 65 years	231 (36.1%)	198 (35.9%)	18 (37.5%)	15 (37.5%)				0.141	0.951
	≧ 70 years	122 (19.1%)	113 (20.5%)	6 (12.5%)	3 (7.5%)				5.461	0.062
	≧ 75 years	42 (6.6%)	39 (7.1%)	2 (4.2%)	1 (2.5%)				1.086	0.638
Height, *cm, M ± Std (Min-Max)*		162.2 ± 7.9 (122.0–185.0)	162.5 ± 8.0 (122.0–185.0)	162.2 ± 7.7 (145.0–176.0)	163.3 ± 7.2 (145.0–176.0)	0.33 [−2.81,3.46]	-0.79 [-4.00,2.42]	-1.11 [-5.44,3.21]	0.217	0.805
Weight, *kg, M ± Std (Min-Max)*		60.8 ± 9.9 (39.0–94.0)	60.9 ± 10.0 (39.0–94.0)	58.6 ± 9.6 (41.0–86.0)	62.0 ± 8.6 (45.0–80.0)	2.26 [-1.31,5.84]	-1.14 [-5.03,2.75]	-3.40 [-8.49,1.69]	1.485	0.227
BMI, *kg/m*^*2*^*, M ± Std (Min-Max)*		22.9 ± 3.1 (15.6–39.0)	23.0 ± 3.2 (15.6–39.0)	21.9 ± 2.7 (17.1–27.8)	23.2 ± 2.8 (17.2–27.9)	1.13 [-0.10,2.36]	-0.20 [-1.47,1.06]	-1.33 [-3.03,0.37]	2.571	0.077
BMI cohorts ^(a)^, *n(%)*	Normal	330 (57.5%)	284 (57.3%)	25 (62.5%)	21 (55.3%)				11.234	0.061
	Underweight	43 (7.5%)	34 (6.9%)	7 (17.5%)	2 (5.3%)					
	Overweight	172 (30.0%)	149 (30.0%)	8 (20.0%)	15 (39.5%)					
	Obese	29 (5.1%)	29 (5.8%)	0 (0.0%)	0 (0.0%)					
Tumor location ^(b)^, *n(%)*	Top	267 (41.7%)	226 (40.9%)	24 (50.0%)	17 (42.5%)				3.153	0.533
	Middle	109 (17.0%)	99 (17.9%)	6 (12.5%)	4 (10.0%)					
	Bottom	264 (41.3%)	227 (41.1%)	18 (37.5%)	19 (47.5%)					
Time of operation, *h, M ± Std*		2.9 ± 1.2	2.9 ± 1.2	3.1 ± 1.3	2.7 ± 1.1	-0.23 [-0.66,0.20]	0.13 [-0.34,0.61]	0.36 [-0.25,0.98]	1.155	0.316

*Abbreviation*: *n =* number, *M* = Mean, *Std* = Standard deviation, *Min =* Minimum value, *Max =* Maximum value, *BMI* = Body mass index

BMI cohorts ^(a)^: Normal: 18.5–23.9 kg/m^2^, Underweight: less than 18.5 kg/m^2^, Overweight: 24.0–27.9 kg/m^2^,Obese: more than 27.9 kg/m^2^

Tumor location ^(b)^: Top: located in and above the duodenum, as in gastric cancer; Middle: located between the duodenum and sigmoid colon, such as transverse colon cancer; Bottom: located at and below the sigmoid colon, such as rectal cancer

### Postoperative VAS

**Fig. 2:**
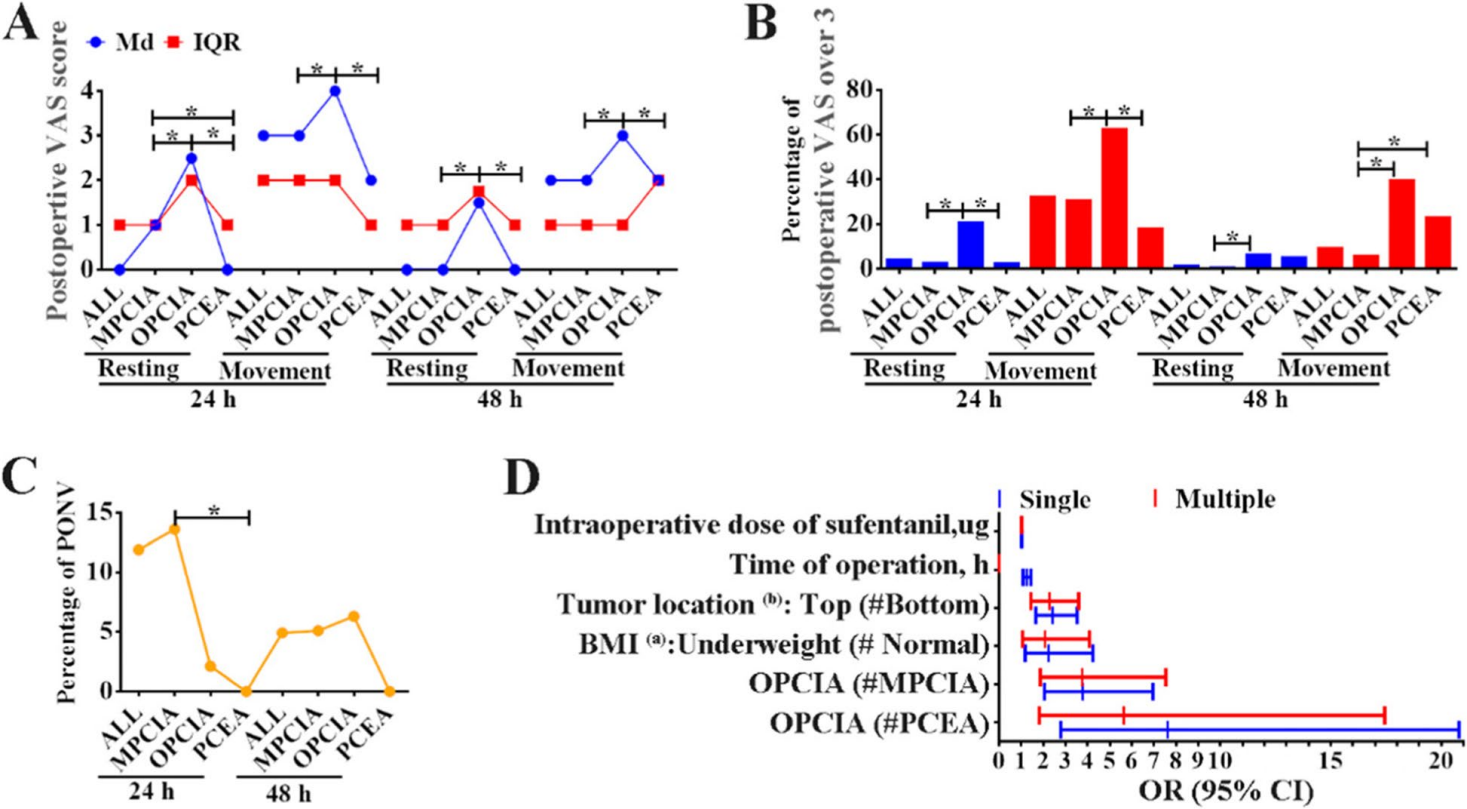
Evaluation of postoperative pain and adverse reactions. *Abbreviation: VAS = Visual analogue score (a range of 0 to 10), Md = Median, IQR = Interquartile range, h = hour(s), PONV = Postoperative nausea and vomiting, BMI = Body mass index.* *: The difference was statistically significant, *p* < 0.05. #: reference variable. BMI ^(a)^: Normal: 18.5–23.9 kg/m^2^, Underweight: less than 18.5 kg/m^2^. Tumor location ^(b)^: Top: located in and above the duodenum, as in gastric cancer; Bottom: located at and below the sigmoid colon, such as rectal cancer

At 24 h after surgery, when the patients were in the resting state, the Md (IQR) of VAS scores of the MPCIA group, OPCIA group and PCEA group were 1 (1), 2.5 (2) and 0 (1), respectively (All *p* < 0.001, Fig. 2A). Among the three groups, 20.8% of OPCIA group had VAS score greater than 3, which was significantly higher than the other two groups (MPCIA: 2.7%, PCEA: 2.5%. *p*_*(OPCIA* VS *MPCIA)*_ < 0.001, *p*_*(OPCIA* VS *PCEA)*_ = 0.01, Fig. 2B). When patients were in the movement state, VAS scores of the three groups from low to high were PCEA group (Md: 2, IQR: 1), MPCIA group (3, 2) and OPCIA group (4, 2) (*p*_*(PCEA* VS *MPCIA)*_ = 0.008, others *p* < 0.001, Fig. 2A). And the proportion of VAS score greater than 3 in OPCIA group was as high as 62.5%, which was significantly higher than the other two groups (MPCIA: 30.6%, PCEA: 17.9%. *p*_*(MPCIA* VS *PCEA)*_ = 0.105, others *p* < 0.001, Fig. 2B).

At 48 h after surgery, VAS scores of OPCIA group (Md and IQR: Resting: 1.5, 1.75. Movement: 3, 1) were significantly higher than those of the other two groups in both resting state (MPCIA: 0,1. PCEA: 0, 1. All *p* < 0.001) and movement state (MPCIA: 2,1. PCEA: 2, 2. *p*_*(OPCIA* VS *MPCIA)*_ < 0.001, *p*_*(OPCIA* VS *PCEA)*_ = 0.002), while there was no statistically significant difference between MPCIA group and PCEA group (Resting: *p* = 0.545, Movement: *p* = 0.264) (Fig. 2A). The percentage of postoperative VAS > 3 in the OPCIA group was the highest (Resting: 6.3%, Movement: 39.6%), followed by the PCEA group (Resting: 5.1%, Movement: 23.1%) and the MPCIA group (Resting: 0.5%, Movement: 5.7%). The differences between the MPCIA group and the OPCIA group in the two states were statistically significant (Resting: *p* = 0.008, Movement: *p* < 0.001), while the differences between the MPCIA group and the PCEA group were only statistically significant in the movement state (Resting: *p* = 0.038, Movement: *p* = 0.001) (Fig. 2B).

### Use of analgesics

The intraoperative dosage of sufentanil in MPCIA group (40.5 ± 14.8 μg) and OPCIA group (36.7 ± 10.9 μg) was significantly higher than that in PCEA group (23.4 ± 7.5 μg) (All *p* < 0.001). And there was no significant difference in the proportion of rescue analgesics used in the three groups on the first day after surgery (MPCIA: 65.9%, OPCIA: 75.0%, PCEA: 75.0%, *p* = 0.265).

### PONV

The incidence of PONV at 24 h after operation in the three groups was from high to low in the MPCIA (13.6%), OPCIA (2.1%) and PCEA (0.0%) groups respectively, and only the difference between the MPCIA group and PCEA group was statistically significant (*p* = 0.006, Fig. 2C). The incidence of PONV in the three groups was 5.1% (MPCIA), 6.3%(OPCIA) and 0.0% (PCEA) at 48 h after operation, and the difference was not statistically significant (*p* = 0.350, Fig. 2C).

### Postoperative recovery

Compared with the MPCIA (69.2 ± 30.1 h) and PCEA (69.9 ± 36.3 h) groups, patients in OPCIA group had the worst postoperative recovery, mainly in the aspects of anal exhaust (93.7 ± 32.2 h, *p*_*(OPCIA* VS *MPCIA)*_ < 0.001, *p*_*(OPCIA* VS *PCEA)*_ = 0.001). Among the three groups, the MPCIA group had the shortest time from the end of surgery to drinking water, about 58.1 ± 35.4 h (PCEA: 78.1 ± 43.4 h, *p*_*(MPCIA* VS *PCEA)*_ = 0.003. OPCIA: 92.7 ± 51.0 h, *p*_*(OPCIA* VS *MPCIA)*_ < 0.001). There was no significant difference in the time between the three groups to get out of bed, remove urinary tube, remove gastric tube, and remove abdominal drainage tube after surgery (Table 2).

**Table 2:** Postoperative recovery

		ALL *n = 640*	MPCIA *n = 552*	OPCIA *n = 48*	PCEA *n = 40*	Difference [95% CI]			Statistical value	*p* value
						MPCIA vs OPCIA	MPCIA vs PCEA	OPCIA vs PCEA		
Time from the end of surgery to the resumption of activity, *h, M ± Std*	First anal exhaust	71.2 ± 31.4	69.2 ± 30.1	93.7 ± 32.2	69.9 ± 36.3	−24.52* [−36.65,-13.39]	−0.64 [−12.75,11.46]	23.88* [8.09,39.67]	14.027	< 0.001*
	Drinking water	62.1 ± 38.7	58.1 ± 35.4	92.7 ± 51.0	78.1 ± 43.4	−34.62* [−53.19,-16.05]	−20.06* [− 37.55,-2.57]	14.56 [−9.96,39.08]	22.779	< 0.001*
	Off-bed activity	54.9 ± 24.7	53.6 ± 23.4	63.9 ± 31.1	61.2 ± 30.9	−10.23 [−21.58,1.11]	−7.60 [− 20.00,4.80]	2.63 [− 13.53,18.79]	5.234	0.006*
	Remove catheter	101.3 ± 41.0	99.8 ± 40.6	108.3 ± 41.3	112.2 ± 44.3	−8.53 [−23.36,6.29]	−12.39 [− 28.52,3.73]	−3.86 [− 24.89,17.17]	2.472	0.085
	Remove abdominal drainage tube	145.7 ± 66.2	142.0 ± 60.4	175.0 ± 103.6	160.8 ± 74.6	−33.03 [−73.31,7.25]	−18.76 [−52.08,14.55]	14.26 [− 36.06,64.60]	5.799	0.003*
	Remove stomach tube	70.1 ± 52.5	70.4 ± 52.2	75.3 ± 56.5	61.3 ± 51.0	−4.88 [−25.51,15.75]	9.13 [−13.76,32.02]	14.01 [−15.75,25.51]	0.693	0.500
Length of postoperative hospital stay, *d, M ± Std*		7.7 ± 3.0	7.6 ± 3.0	8.6 ± 3.5	7.8 ± 2.6	−0.92 [−2.01,0.17]	−0.16 [−1.35,1.03]	0.76 [− 0.79,2.32]	2.059	0.128

*Abbreviation*: *n* = number, *h *= hour(s), *M *= Mean, *Std* = Standard deviation, d = day(s)

*: The difference was statistically significant, *p* < 0.05

The postoperative hospitalization days of patients in the three groups were was 7.6 ± 3.0 d (MPCIA), 8.6 ± 3.5 d (OPCIA) and 7.8 ± 2.6 d (PCEA), respectively, with no statistically significant difference (*p* = 0.128).

### Adverse events

Among the included patients, 2 patients had a second operation, 2 patients had anastomotic fistula, 1 patient had gastrointestinal ulcer, and 2 patients had gastrointestinal bleeding within 1 month after surgery. No adverse events occurred in the PCEA group, 1 anastomotic fistula occurred in the OPCIA group, and the other adverse events were all in the MPCIA group. In addition, one patient had precardiac discomfort (in the MPCIA group) and one patient had episodic atrial fibrillation (in the OPCIA group). There was no significant difference in the incidence of adverse events among groups (*p* = 0.362).

### Correlation analysis

There was a positive correlation between the intraoperative dosage of sufentanil and postoperative VAS scores at 24 h of patients underwent gastrointestinal tumor surgery (Resting: *r* = 0.232, *p* < 0.001. Movement: *r* = 0.186, *p* < 0.001).

### Logistic regression analysis

Univariate regression analysis showed that on the first day after surgery, the factors leading to more severe postoperative pain included the used of OPCIA after surgery (compare with MPCIA and PCEA), BMI less than 18.5 kg/m^2^, gastrointestinal neoplasms in the duodenum and above, longer operation time and more intraoperative sufentanil dosage (Fig. 2D-blue line). The basic characteristics of patients (gender, age, height, weight, BMI, tumor site), operation time, intraoperative dosage sufentanil and postoperative use of PCA type were included for multi-factor analysis. The results showed that the factors leading to moderate-severe postoperative pain were: postoperative use of OPCA (vs MPCIA: OR (95%CI) = 3.758 (1.872–7.542), *p* < 0.001. vs PCEA: OR (95%CI) = 5.636 (1.820–17.446), *p* = 0.003), BMI lower than 18.5 kg/m^2^ (vs BMI of 18.5–23.99 kg/m^2^: OR (95%CI) = 2.081 (1.059–4.089), *p* = 0.033), tumor site in duodenum and above (vs Intestinal tumors of the sigmoid colon and below: OR (95%CI) = 2.280 (1.445–3.596), *p* < 0.001), and large intraoperative dose of sufentanil (OR (95%CI) = 1.017 (1.002–1.031), *p* = 0.021) (Fig. 2D-red line).

### Sensitivity analysis

After PSM, 39 patients were enrolled in MPCIA and OPCIA respectively. The results showed that compared with patients in the OPCIA group, patients in the MPCIA group had lower VAS in the first two days after surgery (either in the resting state or in the movement state), and were able to earlier perform activities after surgery such as anal exhaust and drinking water. In addition, there were no statistically significant difference in the incidence of PONV and other adverse events between the two groups (Supplemental Content Table 1s).

After PSM, 20 patients with MPCIA and 20 patients with PCEA were included. There were no significant differences in postoperative pain assessment, PONV and incidence of adverse events between the two groups by paired analysis. However, compared with MPCIA, patients with PCEA can perform anal exhaust and abdominal drainage tube extraction earlier after surgery (Supplemental Content Table 2s).

## Discussion

In this study, we retrospectively analyzed 640 patients underwent open gastroenteric tumor surgery. Patients were divided into three groups (MPCIA, OPCIA and PCEA) according to the types of PCA used postoperatively. Differences in the characteristics of patients, tumor location, and operation time were not significant. The postoperative analgesia and intestinal function recovery in MPCIA group were similar to those in PCEA group, and significantly better than that in OPCIA group. The incidence of PONV on the first postoperative day in MPCIA group was 13.6%, which was higher than that in PCEA group. However, further sensitivity analysis showed that there was no significant difference in the risk of PONV between the two groups. A total of 9 adverse events occurred within 1 month after surgery, such as secondary surgery, anastomotic fistula, gastrointestinal bleeding, etc., with no statistically significant difference among the three groups. Correlation analysis showed a positive correlation between intraoperative sufentanil dosage and higher postoperative VAS score. Regression analysis showed that BMI, tumor location, intraoperative sufentanil dosage and the type of PCA had influence on postoperative pain.

### The postoperative analgesia effect of sufentanil combined with flurbiprofen ester and dexmedetomidine was better than that of pure opioid

When there was no significant difference in the use of intraoperative sufentanil and postoperative rescue analgesics, VAS scores in the MPCIA group were lower than those in the OPCIA group in the first two days after open gastrointestinal tumor surgery, no matter in the resting or movement state. Furthermore, multifactor binary regression analysis showed that patients using OPCIA had a higher risk of moderate-severe pain on the first day after surgery compared with patients using MPCIA (OR = 3.758, 95%CI: 1.872–7.542). That is, the postoperative analgesia effect of PCIA combined with sufentanil, flurbiprofen ester and dexmedetomidine was better than that of pure opioid PCIA. Flurbiprofen axetil, an effective non-steroidal anti-inflammatory drug (NSAID), can be used in combination with other analgesics for postoperative analgesia to enhance the analgesic effect [15]. Some study found that postoperative administration of flurbiprofen axetil could further decrease the VAS scores, and enhances the analgesic effect of sufentanil [12]. And Gao reported the postoperative VAS scores of dexmedetomidine in combination with sufentanil PCIA were lower than sufentanil PCIA alone [16]. In this study, as adjuvant of multimodal analgesic method, flurbiprofen axetil and dexmedetomidine also improved the postoperative pain management. In addition, the time from the end of surgery to anal exhaust or water intake were both shorter in the MPCIA group than in the OPCIA group. The postoperative recovery of physiological function was closely related to the postoperative analgesic effect [17]. Wang’s research reported that flurbiprofen axetil showed a superior efficacy in early postoperative analgesia, and the recovery time of bowel function was shorter in comparison with the patients using tramadol [18]. Moreover, the well postoperative analgesic methods can dilate gastrointestinal blood vessels, improve microcirculation, promote gastrointestinal metabolism, and promote the recovery of gastrointestinal function [19]. Sufentanil combined with flurbiprofen ester and dexmedetomidine was beneficial for effective analgesia and recovery of intestinal function after open gastrointestinal tumor surgery.

### Sufentanil in combination with flurbiprofen ester and dexmedetomidine was an option for patients who cannot use PCEA for postoperative analgesia

It has been reported that analgesic effect of PCEA was better than OPCIA, and PCEA could effectively reduce the postoperative VAS scores [20–22]. In this study, compared with postoperative analgesia in patients with open gastrointestinal tumors treated with PCEA, patients treated with PCIA with pure opioid configuration had more intraoperative sufentanil dosage and higher postoperative VAS score, leading to a higher risk of moderate to severe postoperative pain (OR = 5.636, 95%CI: 1.820–17.446). The results were similar to previous studies. However, it was worth noting that the use of PCEA had more requirements for patients than PCIA, such as patients should not have coagulation dysfunction [23]. And epidural catheterization may cause total spinal anesthesia and infection [24]. But now, in this study, the proportion of patients using MPCIA who experienced moderate to severe pain in the movement state on the second day after surgery was significantly lower than those using PCEA. And there were no statistically significant differences between the two groups in other states and time. Janice Y Man retrospectively analyzed the pain of patients after minimally invasive reconstruction of pectus infundiformis and showed that the analgesic effect of multi-mode analgesia was comparable to that of thoracic epidural analgesia [25]. Interestingly, in this study, patients in the MPCIA group were able to drink water earlier after surgery than those in the PCEA group. However, further sensitivity analysis showed that anal exhaust and the removal of abdominal drainage tube could be performed earlier in the PCEA group. The review by David Gelman et al. also suggests that some studies have found that epidural analgesia may provide earlier recovery of intestinal function, while some have questioned its role in accelerated recovery after surgery [26]. To sum up, as a multi-mode analgesia method, sufentanil in combination with flurbiprofen ester and dexmedetomidine may be a better option for patients who cannot use PCEA for postoperative analgesia. The difference in postoperative functional recovery between the two methods may require more studies.

### Sufentanil combined with flurbiprofen ester and dexmedetomidine did not increase the incidence of PONV or adverse events in open gastrointestinal tumor surgery

Dong et al. found that the combining dexmedetomidine with sufentanil for PCIA reduced the incidence of nausea/vomiting and improved satisfaction of patients [27]. Additionally, Wen’s research [28] showed that flurbiprofen axetil in combination with small dosage of sufentanil reduced the side effects of postoperative vomiting. However, our results were not completely consistent with previous studies. This may be due to the small sample size of OPCIA group and PCEA group. It is well known that NSAIDs may increase the risk of gastrointestinal bleeding and ulcers after surgery [29]. However, Hongyang Wu et al. performed flurbiprofen for analgesia in 37 cancer patients, and the results showed that the total effective rate was up to 92%, and no side effects such as abdominal pain and gastrointestinal bleeding occurred when using NSAIDS were found [30]. In a multicentre study of 240 patients undergoing upper abdominal surgery, flurbiprofen was superior to tramadol in early postoperative analgesia with a significantly lower incidence of adverse reactions [18]. These results were similar to the no significant increase in the rate of adverse events in the MPCIA group compared to the other two groups in the study. In other words, sufentanil in combination with flurbiprofen ester and dexmedetomidine was safe for postoperative analgesia in patients undergoing surgery for gastrointestinal tumors.

### Factors affecting postoperative analgesia

Correlation analysis showed that there was a weak positive correlation between intraoperative sufentanil dosage and VAS score on the first postoperative day (Resting: *r* = 0.232. Movement: *r* = 0.186). Regression analysis further confirmed the effect of intraoperative sufentanil on postoperative analgesia (OR = 1.017, 95%CI: 1.002–1.031). Patricia Lavand ‘Homme et al., in their review, showed that in perioperative setting, intraoperative administration of high doses of opioids increases postoperative opioid requirements and worsens pain scores (acute tolerance or perioperative “opioid-induced hyperalgesia”) [31]. Eric Albrecht et al., in an analysis of 27 randomized controlled studies, demonstrated low evidential certainty that large intraoperative opioid doses increased postoperative pain scores [32]. These results are similar to our results, but more studies are needed to explore the effects of intraoperative opioid dosage and postoperative analgesia.

In this study, regression analysis results showed that tumor location above duodenum (vs Intestinal tumors of the sigmoid colon and below, OR = 2.280, 95% CI: 1.445–3.596) had greater influence on postoperative analgesia. Previous studies have reported that postoperative pain was related to tumor location in the lower third of the stomach [33]. Similar findings have been confirmed by other studies [34]. It was noteworthy that, compared with patients with normal BMI (18.5–23.9 kg/m^2^), a higher BMI has no effect on postoperative analgesia, while a lower BMI (less than 18.5 kg/m^2^) was the influencing factor for poor postoperative analgesia (OR = 2.081, 95% CI: 1.059–4.089). Conhen B et al., in an analysis of 808 pediatric inpatients who had undergone non-cardiac surgery, confirmed that there was no clinically important increase in pain scores or opioid consumption in association with higher BMI [35]. And Xue-ying Lai et al. found that a lower BMI was an independent risk factor for pain during colonoscopy [36]. Furthermore, it was confirmed that patients with BMIs of less than 18.5 kg/m^2^ had worse analgesia on the first day after surgery in our subsequent study [37].

### Strengths and limitations

In this study, we combined sufentanil, flurbiprofen ester and dexmedetomidine to configure PCIA for postoperative analgesia. It was proved the first time that the combined effect of these three drugs for postoperative analgesia was comparable to that of PCEA, significantly superior to pure opioid-PCIA, and did not increase the incidence of gastrointestinal bleeding and other adverse events. This will be an option for patients who cannot use PCEA to provide postoperative analgesia. Through correlation analysis and regression analysis, we determined that intraoperative sufentanil dosage, lower BMI and higher gastrointestinal tumor location had an impact on postoperative analgesia. These will provide some guidance for optimizing postoperative analgesia in the future. However, this study was a retrospective study and the confounding bias pose a greatest risk to our study. Therefore, we performed sensitivity analysis through PSM to support our analysis. In addition, the results were based only on the analgesic methods we use and may not be generalized. More prospective studies need to be conducted to enhance the reliability of the results of this study. Furthermore, we only compared this multi-mode analgesia with epidural analgesia and traditional pure opioid analgesia. Whether MPCIA was definitely better than the combination of flurbiprofen and sufentanil or the combination of dexmedetomidine and sufentanil, which needs to be verified by further study.

## Conclusions

The multimodal PCIA (PCIA with sufentanil combined with dexmedetomidine and flurbiprofen) effectively improved the postoperative pain after open gastroenteric tumor surgery, which was comparable to that of PCEA, significantly superior to pure opioid-PCIA. This will be an option for patients who cannot use PCEA to provide postoperative analgesia. More intraoperative sufentanil dosage, lower BMI and higher gastrointestinal tumor location had an impact on postoperative analgesia, which will provide some guidance for optimizing postoperative analgesia in the future.

## Supplementary Information

**Additional file 1: Table 1s**. Comparison between MPCIA and OPCIA. **Table 2s**. Comparison between MPCIA and OPCIA.

## Data Availability

The datasets used and analyzed during the current study are available from the corresponding author on reasonable request.

## Abbreviations

PCIA: Patient-controlled intravenous analgesia
PCEA: Patient-controlled epidural analgesia
MPCIA: PCIA with sufentanil, flurbiprofen axetil, dexmedetomidine and metoclopramide
OPCIA: PCIA with sufentanil, tramadol and metoclopramide
PCEA: PCEA with sufentanil and ropivacaine
VAS: Visual analogue scale
OR: Odds ratio
95% CI: 95% Confidence interval
NSAID: Non-steroidal anti-inflammatory drug
