# Supplementary material for: Analgesic effects of sufentanil in combination with flurbiprofen axetil and dexmedetomidine after open gastrointestinal tumor surgery: a retrospective study
Source: BMC Anesthesiol. 2022 Apr 29;22:130. doi: 10.1186/s12871-022-01670-0 (PMC9052469; doi:10.1186/s12871-022-01670-0)
Supplement: Supplementary file 1 — Additional file 1: Table 1s. Comparison between MPCIA and OPCIA. Table 2s. Comparison between MPCIA and OPCIA. [file 12871_2022_1670_MOESM1_ESM.docx]

**Supplemental Content**

**Table 1s. Comparison between MPCIA and OPCIA**

|  | | MPCIA n=39 | OPCIA n=39 | Difference (95%CI) | Statistical value | p value |
| --- | --- | --- | --- | --- | --- | --- |
|  |  |  |  |  |  |  |
| Gender, n(%) | Male | 22(56.4%) | 22(56.4%) | / | / | 1.000 |
|  | Female | 17(43.6%) | 17(43.6%) |  |  |  |
| Age, year-old, M±Std | | 60.2±12.1 | 59.5±9.3 | 0.72 (-4.46,5.90) | 0.280 | 0.781 |
| Height, cm, M±Std | | 162.8±9.4 | 162.5±7.6 | 0.31 (-3.74,4.36) | 0.154 | 0.879 |
| Weight, kg, M±Std | | 60.4±13.0 | 58.1±9.7 | 2.31 (-3.07,7.69) | 0.869 | 0.390 |
| BMI, kg/m2, M±Std | | 22.7±3.9 | 21.9±2.7 | 0.74 (-0.62,2.09) | 1.100 | 0.278 |
| Tumor location (b), n(%) | Top | 17(43.6%) | 21(53.8%) | / | 5.571 | 0.134 |
|  | Middle | 6(15.4%) | 4(10.3%) |  |  |  |
|  | Bottom | 16(41.0%) | 14(35.9%) |  |  |  |
| Time of operation, h, M±Std | | 3.2±1.0 | 3.1±1.3 | 0.07 (0.27,-0.47) | 0.276 | 0.784 |
| Intraoperative dose of sufentanil, ug, M±Std | | 35.9±8.6 | 35.3±7.6 | 0.55 (1.93,-2.25) | 0.285 | 0.777 |
| VAS at 24 h (Resting) | Md, IQR | 1, 1 | 3, 2 | / | -3.955 | <0.001* |
|  | VAS>3, n(%) | 0(0.0%) | 9(23.1%) | / | / | 0.002* |
| VAS at 24 h (Movement) | Md, IQR | 3, 2 | 4, 2 | / | -3.178 | 0.001* |
|  | VAS>3, n(%) | 10(25.6%) | 24(61.5%) | / | / | 0.003* |
| Use of rescue analgesics, n(%) | | 24(51.5%) | 29(74.4%) | / | / | 0.302 |
| VAS at 48 h (Resting) | Md, IQR | 1, 1 | 2, 2 | / | -4.144 | <0.001* |
|  | VAS>3, n(%) | 0(0.0%) | 3(7.9%) | / | / | 0.240 |
| VAS at 48 h (Movement) | Md, IQR | 2, 1 | 3, 1 | / | -4.510 | <0.001* |
|  | VAS>3, n(%) | 2(5.3%) | 17(43.6%) | / | / | <0.001* |
| PONV, n(%) | at 24 h | 4(10.3%) | 1(2.6%) | / | / | 0.358 |
|  | at 48 h | 0(0.0%) | 3(7.7%) | / | / | 0.240 |
| Time from the end of surgery to the resumption of activity, h, M±Std | Anal exhaust | 70.2±24.1 | 97.8±34.7 | -27.58 (-42.92,-12,25) | -3.664 | 0.001* |
|  | Drinking water | 61.7±31.3 | 98.1±53.0 | -36.39 (-55.87,-16.91) | -3.797 | 0.001* |
|  | Off-bed activity | 62.1±24.3 | 65.4±32.0 | -3.30 (-15.57,8.96) | -0.546 | 0.588 |
|  | Withdrawal of catheter | 106.5±37.2 | 102.0±41.8 | 4.47 (-14.09,23.04) | 0.490 | 0.628 |
|  | Remove abdominal drainage tube | 153.0±53.7 | 181.6±116.3 | -28.59 (-74.99,17.81) | -1.264 | 0.217 |
|  | Remove stomach tube | 118.0±84.8 | 110.6±40.4 | 7.40 (-55.19,69.99) | 0.255 | 0.802 |
| Adverse reactions within one month after surgery, n(%) | Reoperation | 0(0.0%) | 0(0.0%) | / | / | / |
|  | Anastomotic fistula | 0(0.0%) | 1(2.6%) | / | / | 1.000 |
|  | Peptic ulcer | 0(0.0%) | 0(0.0%) | / | / | / |
|  | Gastrointestinal bleeding | 1(2.6%) | 0(0.0%) | / | / | 1.000 |
|  | Cardiovascular disease | 0(0.0%) | 1(2.6%) | / | / | 1.000 |
| Length of postoperative hospital stay, d, M±Std | | 7.7±2.5 | 8.88±3.6 | -1.08 (-2.50,0.34) | -1.536 | 0.133 |

***Abbreviation:*** *n=number, M=Mean, Std=Standard deviation, BMI=Body mass index, VAS=Visual analogue score (a range of 0 to 10), h = hour(s), Md=Median, IQR=Interquartile range. PONV = Postoperative nausea and vomiting, d = day(s). *: The difference was statistically significant, p < 0.05.*

***BMI cohorts^(a)^****: Normal: 18.5-23.9 kg/m^2^, Underweight: less than 18.5 kg/m^2^, Overweight:24.0-27.9 kg/m^2^,Obese: more than 27.9 kg/m^2^.*

***Tumor location^(b)^****: Top: located in and above the duodenum, as in gastric cancer; Middle: located between the duodenum and sigmoid colon, such as transverse colon cancer; Bottom: located at and below the sigmoid colon, such as rectal cancer.*

**Table 2s. Comparison between MPCIA and OPCIA**

|  | | MPCIA n=20 | PCEA n=20 | Difference (95%CI) | Statistical value | p value |
| --- | --- | --- | --- | --- | --- | --- |
|  |  |  |  |  |  |  |
| Gender, n(%) | Male | 11(55.0%) | 12(60.0%) | / | / | 1.000 |
|  | Female | 9(45.0%) | 8(40.0%) |  |  |  |
| Age, year-old, M±Std | | 57.7±12.8 | 57.7±12.2 | 0.00 (-7.71,7.71) | 0.000 | 1.000 |
| Height, cm, M±Std | | 165.5±9.8 | 163.6±7.7 | 1.85 (-3.88,7.58) | 0.676 | 0.507 |
| Weight, kg, M±Std | | 63.3±10.5 | 61.7±10.8 | 1.63 (-5.69,8.94) | 0.465 | 0.647 |
| BMI, kg/m2, M±Std | | 23.1±3.0 | 23.0±3.2 | 0.12 (-2.12,2.35) | 0.109 | 0.914 |
| Tumor location (b), n(%) | Top | 7(35.0%) | 8(40.0%) | / | 0.564 | 0.828 |
|  | Middle | 2(10.0%) | 3(15.0%) |  |  |  |
|  | Bottom | 11(55.0%) | 9(45.0%) |  |  |  |
| Time of operation, h, M±Std | | 2.9±1.3 | 2.6±1.1 | 0.31 (-0.40,1.02) | 0.908 | 0.375 |
| Intraoperative dose of sufentanil, ug, M±Std | | 30.9±11.8 | 28.0±5.5 | 2.90 (-2.44,8.24) | 1.136 | 0.270 |
| VAS at 24 h (Resting) | Md, IQR | 1, 2 | 0, 2 | / | -1.936 | 0.053 |
|  | VAS>3, n(%) | 0(0.0%) | 0(0.0%) | / | / | / |
| VAS at 24 h (Movement) | Md, IQR | 2.5, 2 | 2, 2.25 | / | -0.670 | 0.503 |
|  | VAS>3, n(%) | 7(35.0%) | 5(25.0%) | / | / | 0.731 |
| Use of rescue analgesics, n(%) | | 12(60.0%) | 16(80.0%) | / | / | 0.301 |
| VAS at 48 h (Resting) | Md, IQR | 0, 1 | 0, 2 | / | -0.264 | 0.792 |
|  | VAS>3, n(%) | 0(0.0%) | 0(0.0%) | / | / | / |
| VAS at 48 h (Movement) | Md, IQR | 1.5, 2 | 2, 2.25 | / | -0.041 | 0.968 |
|  | VAS>3, n(%) | 3(15.8%) | 3(15.8%) | / | / | 1.000 |
| PONV, n(%) | at 24 h | 3(15.0%) | 0(0.0%) | / | / | 0.231 |
|  | at 48 h | 0(0.0%) | 0(0.0%) | / | / | / |
| Time from the end of surgery to the resumption of activity, h, M±Std | Anal exhaust | 71.4±23.9 | 56.6±23.7 | 14.82 (0.73,28.90) | 2.229 | 0.040* |
|  | Drinking water | 64.1±33.2 | 62.1±31.0 | 1.97 (-26.69,30.64) | 0.146 | 0.886 |
|  | Off-bed activity | 50.7±20.9 | 63.0±25.2 | -12.29 (6.11,--25.18) | -0.201 | 0.060 |
|  | Withdrawal of catheter | 130.3±57.0 | 115.1±51.8 | 15.16 (-32.56,62.88) | 0.677 | 0.509 |
|  | Remove abdominal drainage tube | 175.4±87.0 | 148.0±48.4 | 27.41 (-55.76, 110.58) | 0.760 | 0.469 |
|  | Remove stomach tube | 101.8±69.9 | 45.6±28.7 | 56.19 (10.95,101.43) | 2.810 | 0.020* |
| Adverse reactions within one month after surgery, n(%) | Reoperation | 0(0.0%) | 0(0.0%) | / | / | / |
|  | Anastomotic fistula | 0(0.0%) | 0(0.0%) | / | / | / |
|  | Peptic ulcer | 0(0.0%) | 0(0.0%) | / | / | / |
|  | Gastrointestinal bleeding | 1(5.0%) | 0(0.0%) | / | / | 1.000 |
|  | Cardiovascular disease | 0(0.0%) | 0(0.0%) | / | / | / |
| Length of postoperative hospital stay, d, M±Std | | 8.0±3.3 | 7.3±2.6 | 0.65 (-1.54,2.84) | 0.622 | 0.541 |

***Abbreviation:*** *n=number, M=Mean, Std=Standard deviation, BMI=Body mass index, VAS=Visual analogue score (a range of 0 to 10), h = hour(s), Md=Median, IQR=Interquartile range. PONV = Postoperative nausea and vomiting, d = day(s). *: The difference was statistically significant, p < 0.05.*

***BMI cohorts^(a)^****: Normal: 18.5-23.9 kg/m^2^, Underweight: less than 18.5 kg/m^2^, Overweight:24.0-27.9 kg/m^2^,Obese: more than 27.9 kg/m^2^.*

***Tumor location^(b)^****: Top: located in and above the duodenum, as in gastric cancer; Middle: located between the duodenum and sigmoid colon, such as transverse colon cancer; Bottom: located at and below the sigmoid colon, such as rectal cancer.*
